# Improving quality of antenatal care services in a high case load tertiary care teaching hospital (Ganesh Shankar Vidyarthi Memorial (GSVM) Medical College) in Kanpur, Uttar Pradesh, India

**DOI:** 10.1136/bmjoq-2021-001446

**Published:** 2022-05-10

**Authors:** Praveen Kumar Sharma, Sebanti Ghosh, Vikrant Prabhakar, Mahtab Singh, Thomas Forissier, Rajendra Prasad, Anjali Datta, Benazir Patil, Kiran Pandey, Neena Gupta, Seema Nigam

**Affiliations:** 1 Alive & Thrive, FHI 360 India, New Delhi, Delhi, India; 2 Department of Community Medicine, Adesh Medical College & Hospital, Kurukshetra, Haryana, India; 3 Department of Obstetrics and Gynecology, Ganesh Shankar Vidyarthi Memorial Medical College, Kanpur, Uttar Pradesh, India; 4 Department of Community Medicine, Ganesh Shankar Vidyarthi Memorial Medical College, Kanpur, Uttar Pradesh, India

**Keywords:** quality improvement, quality improvement methodologies, women's health, healthcare quality improvement

## Abstract

In India, half of all pregnant women between the ages of 15 and 49 years are anaemic. In Uttar Pradesh (UP), this figure is slightly higher at 51%. Unfortunately, only 5.4% pregnant women received full antenatal care (ANC) (National Family Health Survey 4, 2015–2016). A formative research conducted in UP in 2016 found that only 9% of pregnant women in UP consume the five recommended food groups, as per global recommendations.

Ganesh Shankar Vidyarthi Memorial Medical College Hospital is one of the four high case load tertiary care facilities in Kanpur, UP, with an obstetrics and gynaecology (OBGY) outpatient department (OPD) of 2500–3000 consultations with delivery load of 250–300 deliveries per month and paediatric OPD of approximately 5400–6000 consultations per month. It was identified that pregnant women visiting the OPD for ANC were not receiving maternal nutrition-related services, and anthropometric measurements to assess nutritional status and gestational weight gain were also not done.

The department of OBGY decided to apply the four-step Point of Care Quality Improvement (POCQI) approach using Plan–Do–Study–Act cycle for implementation of the maternal nutrition protocol during ANC.

In April 2019, with the support of A&T, the hospital team applied the POCQI methodology to improve ANC service provision. By the end of 2019, the measurement and recording of anthropometric parameters increased to 84% and 74% for height and weight, respectively, from the baseline of zero. Hb testing increased from 58% to 84% and blood pressure (BP) monitoring from zero to 84%. Maternal nutrition counselling was delivered to 76% of the pregnant women visiting the OPD, which was a significant achievement for a new practice introduced into the system.

The improved practices identified and implemented by the department are being sustained through active engagement of the staff and supportive leadership of the department of OBGY.

## Problem

As per National Family Health Survey 4 (2015–2016), 50% of pregnant women between the age of 15 and 49 years are anaemic in the country, and Uttar Pradesh (UP) stands close at 51%. Nationally, only 30% of pregnant women consume 100 or more iron folic acid (IFA) tablets during pregnancy, and UP has a dismal IFA consumption rate of 13% as per the NFHS 4 data. A formative research conducted in 2016 in UP showed that only 9% of pregnant women consume foods from at least five recommended food groups, as per global recommendation pertaining to maternal diet diversity.^1^

Ganesh Shankar Vidyarthi Memorial (GSVM) Medical College Kanpur is a state-run medical college in the city of Kanpur, UP, affiliated to Chhatrapati Shahu Ji Maharaj University, Kanpur. It is a 1055-bed hospital with 120 beds dedicated to the department of paediatric and 235 beds in the obstetrics and gynaecology (OBGY) department. It has a team of 18 faculty and senior doctors, 48 residents and 34 staff nurses in OBGY department and 8 faculty and senior doctors, 37 residents and 31 staff nurses in paediatric department. Being a tertiary care facility, it caters to patients from Kanpur and adjoining districts of Kanpur Dehat, Unnao, Hamirpur and Fatehpur. In any given month, an average of 2700 women attend the OBGY outpatient department (OPD) out of which 325 undergo antenatal care (ANC) check-ups and 270 deliveries take place per month. Antenatal maternal nutrition (MN) services were missing from the current package of ANC services provided in the hospital. As per available records, approximately 40% of pregnant women missed out on haemoglobin (Hb) testing for assessing anaemia status. Gaps identified in nutrition assessment (height, weight measurement, and calculation of body mass index) and nutritional counselling on diet and IFA and calcium supplementation was not up to the mark, and hence, this needed to be prioritised and integrated into the ANC service provision.

Recognising the importance of MN for positive maternal health and pregnancy outcome as well as the foundation for health and well-being of the newborn, the department of OBGY decided to apply the four-step Point of Care Quality Improvement (POCQI) approach^2^ for implementation of the MN protocol during ANC.

A Quality Improvement team was formed with members from department of OBGY and preventive and social medicine (PSM) comprising of the heads of the two departments, faculty doctors, postgraduate resident doctors and interns.

The antenatal visits include history taking of the pregnant women, anthropometric measurement like height and weight measurement, BP check, laboratory investigations like Hb, oral glucose tolerance test, IFA and calcium supplementation, administration of tetanus toxoid injection, counselling on diet, rest, self-care, importance of institutional delivery, early and exclusive breast feeding and micro birth planning (Guidelines for antenatal care and skilled attendance at birth by auxiliary nurse midwives (ANMs)/LHVs/SNs (MOHFW). The team chose to focus on improving anthropometric measurements, Hb testing, measurement of BP and MN counselling during ANC and developed a Specific, Measurable, Achievable, Relevant, Time bound (SMART) aim.

SMART Aim Statement was formulated to improve MN services and counselling during ANC at GSVM Medical College by:

- Increasing measurement of height and weight and nutrition counselling among pregnant women (PW) from 0% to 70% over 5 months.
- Increasing Hb measurement among pregnant women from 58% to 70% over 5 months.

## Background

Pregnancy is a time of intensified nutritional vulnerability, and the nutritional status of women before and during pregnancy can have a substantial influence on fetal and maternal outcomes. Fetal growth restriction is one of the leading causes of stunting worldwide.^3^ The relationship between nutritional status and health of mothers and new born is well documented. ANC is the key entry point for pregnant women to receive a broad range of health promotion and prevention services. Government of India recommends a minimum of four ANC visits, ideally at 16, 24–28, 32 and 36 weeks and recommends health promotion including nutrition counselling as one of its important components.^4^ It has been shown in several developing countries that women attending regular ANC exhibit better knowledge, attitudes and antenatal practices compared with those not availing ANC.^5 6^

India has been investing in regular in-service training to improve clinical practices in ANC and care around birth.^7^ The WHO recommends an effective balance between public health interventions and clinical services through alignment of medical education system with health system needs.^8^ India’s national nutrition strategy also suggests engagement of educational institutes including medical colleges for addressing malnutrition.^9^ Maternal Infant & Young Child Nutrition is often not prioritised in service delivery in associated hospitals of medical colleges. Moreover, quality of care for maternal, infant and young child health and nutrition services has remained suboptimal. Globally, poor quality of service delivery has emerged as an important driver of amenable mortality across conditions, contributing 61% of neonatal conditions and half of deaths from maternal causes. Poor quality of care results in higher number of deaths from treatable causes compared with non-utilisation.^10^

## Measurement

There was no register to record data on ANC service provision. Other than Hb test results, no other data or register existed to determine if a PW had received a comprehensive package of ANC services, including MN services and counselling, as per global and national guidelines. The QI team thus agreed to take the baseline for weight, height measurement and BP check at zero. As part of the changes introduced, one of the staff nurses started documenting services provided to PW attending the ANC OPD. A register was maintained to record height, weight, Hb, BP and counselling details of ANC clients. Later department got a specifically designed register printed to record ANC details. The data were collated on a monthly basis for analysis. Additionally, ANC services were mentioned on mother and child protection (MCP) card to track services provided to PW.

The QI team assigned a senior resident with the responsibility of verifying whether a PW had received all ANC services (eg, anthropometric measurement, BP check, Hb testing) before she was directed to the faculty doctor for a review of the findings and an obstetric assessment. QI team also started developing time series chart of all services being provided to track the improvement over time.

## Design

**Figure 1:**
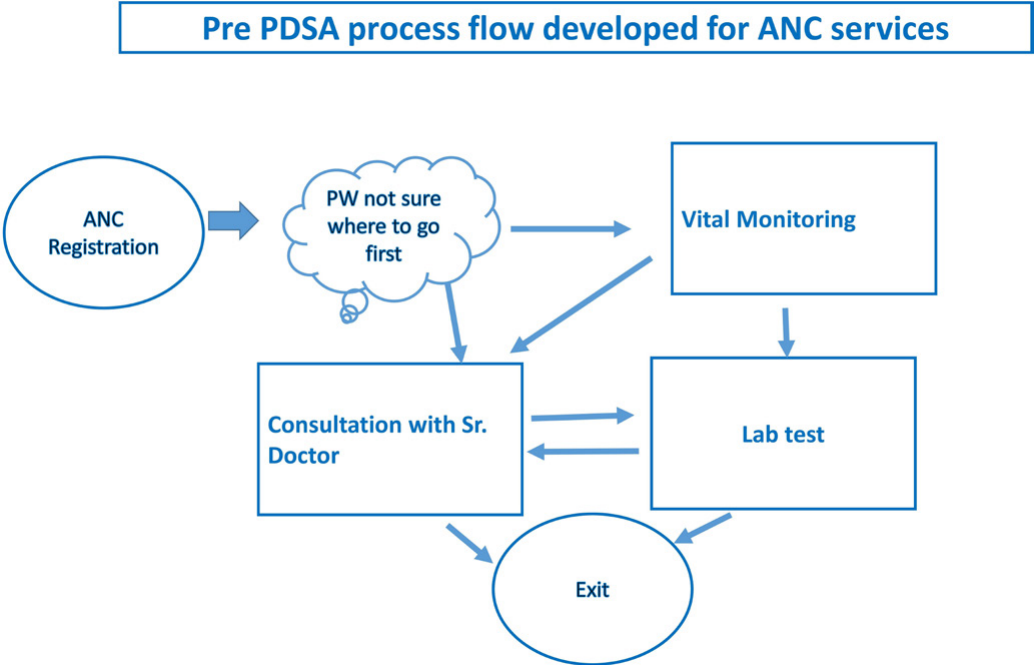
Pre-PDSA original process flow. ANC, antenatal care; PDSA, Plan–Do–Study–Act; PW, pregnant women.

In order to understand the current delivery process of ANC for PW and identify gaps if any, the QI team tried to map existing processes from the time a PW entered the OPD to the time she exited using a flow chart. The team found that there was no fixed process for PW to get services as few of PW were going for vital monitoring and some of them were going directly to senior doctor for consultation. Many PW were missing Hb test and counselling as they we leaving hospital just after consultation with senior doctor (figure 1). Process mapping exercise revealed two critical gaps. First, there was no systematic patient flow management mechanism in place to ensure that all PW who entered the OPD received all the ANC services that were provided. As a result, some women did not avail all components of essential ANC services. Second, a number of key MN services, anthropometric measurement and nutrition counselling were not being provided. The QI team decided to undertake the following steps for strengthening ANC services including MN: (A) implementing a systematic patient flow management system; (B) ensuring delivery of all key MN services during ANC following standard protocols with focus on anthropometric measurement for nutrition status assessment, Hb testing, provision of IFA and calcium supplementation and counselling; (C) engaging HIV counsellor to strengthen MN counselling; and (d) implementing a system for recording the services provided to PW during ANC visit. The QI team assumed that these interventions will help fill the gap identified during process mapping.

## Strategy

The QI team applied the four steps of POCQI approach and identified the problem through process mapping; QI team also applied various changes related to ANC services and counselling and decided to use Plan–Do–Study–Act (PDSA) cycle for each change made in three units of the OBGY department to test it whether it is feasible to do in the local context and will it help in improving the services. Required adjustments were to be done as per test findings.

### Planning the key changes

A decision to test the following changes to strengthen ANC services comprised: (A) implementing a systematic patient flow management system; (B) ensuring delivery of all essential MN services during ANC; (C) engaging HIV counsellor for MN counselling; and (D) developing an ANC register where all services a woman received during ANC could be recorded

### Doing the tests

Table 1 provides details on the change processes that the QI team developed and tested on a small scale.

**Table 1:** Details of changes tested

PDSA	Categories of changes	Plan	Do	Study	Act
PDSA 1 May 2019, week 1	Patient flow management	A team of OPD doctors, nurses and counsellors developed a plan for improved patient flow during ANC that allowed the pregnant women to obtain various ANC service components sequentially. Volunteers from the Red Cross Society were engaged to support in crowd management and systematically direct the patient flow.	This ‘change’ was tested in three ANC OPDs over 3 days covering 30 ANC clients.	This change was successful, and team was able to manage crowd	*This change was found feasible and was then applied to all the OPDs and adopted.*
PDSA 2 May 2019, week 2	Recording and verification of ANC services received	The QI team assigned a senior resident with the responsibility of verifying whether a PW received all ANC services (eg, anthropometric measurement, BP check and Hb testing) before the patient was directed to the senior faculty member/doctor for a review of the findings and an obstetric assessment.	This ‘change’ was tested in three ANC OPDs over 3 days covering 36 ANC clients.	This change was found to be effective in ensuring that pregnant women received all the available ANC services but felt difficult to write each service.	*Department printed an ANC register to record all the services during the OPD and then change was adopted.*
PDSA 3 May 2019, week 3	Ensuring receipt of Hb test report on the same day	As per current practice, PW did not receive their Hb test results on the same day. Instead, they had to make another OPD visit so that the doctor could review and discuss their findings with them. Often PW, especially those who lived far from the hospital, did not return for this second visit, given their time and financial constraints. The QI team decided to try to make the Hb report available on the same day as it was critical for timely assessment of anaemia and treatment.	This ‘change’ was tested in three ANC OPDs over 2 days covering 19 ANC clients.	*The practice was not found feasible*	This change could not be applied in 100% of the cases due to the limited capacity of the in-house laboratory. *This change did not work and was abandoned*.
PDSA 4 June 2019, week 1	Use of MCP card to record and track ANC data	1. The QI team facilitated the availability and use of government-issued MCP cards, which record and track anthropometric measurements, clinical examination (BP) and tests (eg, blood test report for Hb, urine test for sugar and albumin). Initially, a junior resident (JR) doctor was assigned the task to enter the ANC data for each pregnant woman from the OPD slips to the MCP cards. 2. The team decided to assign the responsibility to the auxiliary nurse midwife (ANM) who worked at the adjoining family planning centre.	1. This ‘change’ was tested in three ANC OPDs over 2 days, covering 18 ANC clients by JR. 2. Tested in three ANC OPDs over 3 days by ANM covering 35 ANC clients.	1. The team found that this was not sustainable since the JR was also responsible for other patients in the OPD. 2. The team found that not all ANC clients reported to the ANM to get their data from the OPD slip transferred to their MCP card. Also, in some cases, the data related to weight, height and BP was not recorded on the OPD slip.	The QI team briefed and oriented the JRs and the ANM on the importance of recording these parameters in the OPD slips and the MCP cards. Hospital management made JR and ANM (rotation wise) responsible for this work and included it as one of the their key responsibility. QI team was able to obtain expected results by this change and decided to adopt the practice.
PDSA 5 May 2019, week 4	Nutritional counselling	1. The hospital did not have a dedicated nutrition counsellor and hence the HIV counsellor was assigned the additional responsibility of counselling the pregnant women on the importance of a healthy diet. 2. Additionally, a junior doctor from the OBGY department was assigned the responsibility of nutrition counselling.	1. This ‘change’ was tested in three ANC OPDs over 3 days covering 30 ANC clients with HIV counsellor. 2. Tested three ANC OPDs over 3 days covering 36 ANC clients with junior doctor and HIV counsellor.	1-However, due to the high patient load, this was not sustainable. Group counselling was also not a feasible option since there was not enough space in the OPD. 2. It worked well with additional junior doctor.	Women were classified based on their Hb status into normal, moderate and severely anaemic. Need-based counselling was provided to moderately and severely anaemic PW. Changes adapted, tested further and then adopted.
PDSA 6 May 2019, week 4	Assessment of quality of counselling	To assess the quality of nutrition counselling and to improve counselling, the departments of OBGY and PSM developed an exit interview questionnaire for pregnant women attending OPD.	This ‘change’ was tested in one ANC OPDs over 1 day covering 12 ANC clients.	A junior resident from the department of PSM was assigned to conduct the interviews with randomly chosen patients twice a week.	The process was found to be both feasible and sustainable. Change was adopted.
PDSA 7 June 2019, week 2	Developing data recording system	The QI team introduced the system of recording ANC data, including maternal nutrition services. A staff nurse assigned to record the data.	This ‘change’ was tested in two ANC OPDs over 2 days covering 22 ANC clients.	Staff nurse was able to record ANC data of maternal nutrition services provided to PW.	Based on the suggestion of the QI team, the OBGY department developed a new register to record all relevant information of services provided to pregnant women in the ANC OPD and change was adopted.

ANC, antenatal care; BP, blood pressure; Hb, haemoglobin; MCP, mother and child protection; OBGY, obstetrics and gynaecology; OPD, outpatient department; PW, pregnant women.

### Studying the change process

The QI team found that during the testing phase, most of the changes listed in table 1 could be feasibly scaled except the same-day availability of Hb test reports, which continues to be a challenge due to the limited capacity of the in-house laboratory testing.

### Acting on the study findings

The changes did not require additional resources nor did it add to the already existing workload of the doctors and staff nurses in the OBGY department. All units of the OBGY department adopted the successful changes based on the learnings of this QI project.

QI team ran multiple PDSA in May and June and developed change bundle that was found to be feasible to scale except same day availability of Hb test reports. Only a limited number of reports were available, and it continues to remain a challenge requiring interventions that are beyond the scope of this project. However, the rest of the changes did not require any additional resources nor added to the workload of the doctors and staff nurses in the OBGY department. On the recommendation of the QI team, it was therefore decided to adopt the changes in all the units of the OBGY department.

All the staff nurse and doctors working in other units and in different shifts were oriented on the successful changes that were implemented. A circular has been issued by the head of the department to institutionalise these changes as part of the system to provide quality ANC services. Register for recording the services helped in measuring and tracking the services provided to the client. Introduction of MCP card was helpful in reconfirming the services provided, and it is also helpful for PW to have the records of services she received.

## Result

**Figure 2:**
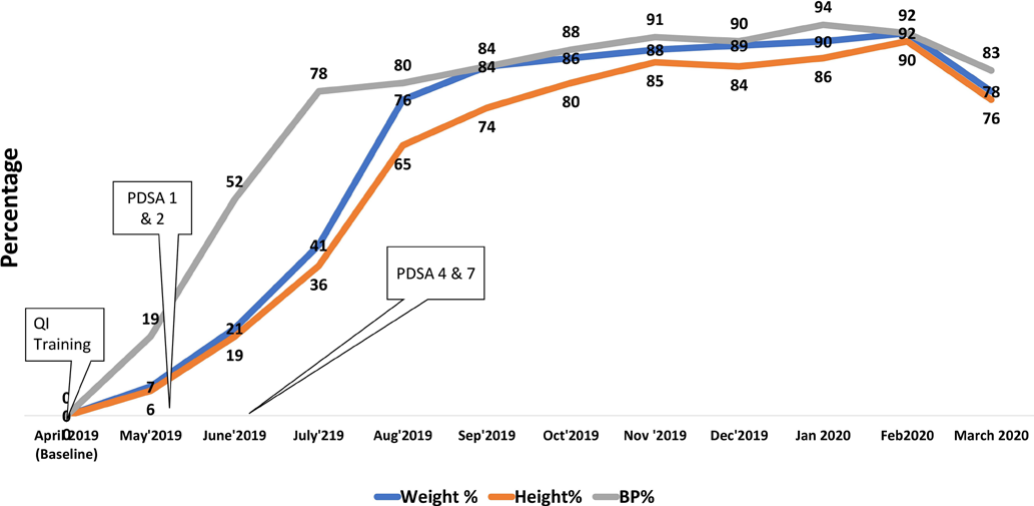
Improvements in ANC services. ANC, antenatal care; BP, blood pressure; PDSA, Plan–Do–Study–Act.

**Figure 3:**
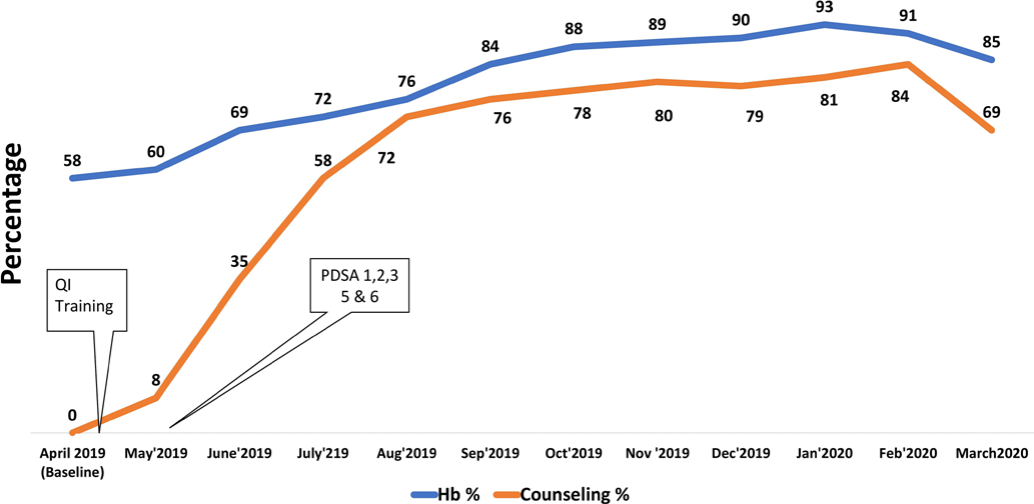
Improvements in ANC services. ANC, antenatal care; Hb, haemoglobin; MN, maternal nutrition; PDSA, Plan–Do–Study–Act.

After implementing the POCQI approach and testing changes over a duration of 5 months, more number of pregnant women were now being provided MN-related ANC services (figures 2 and 3). Further is a summary of the results: (A) the percentage of pregnant women weighed during ANC increased from 0% in April to 84% in September 2019 and height measurement increased from 0% to 74%; (B) BP measurement increased from 0% to 84%; (C) Hb testing increased from 58% at baseline to 84% in September 2019; (D) MN counselling was provided to 76% of the pregnant women visiting the ANC OPD in September 2019, a significant achievement as this was a new practice introduced in the OPD.

**Figure 4:**
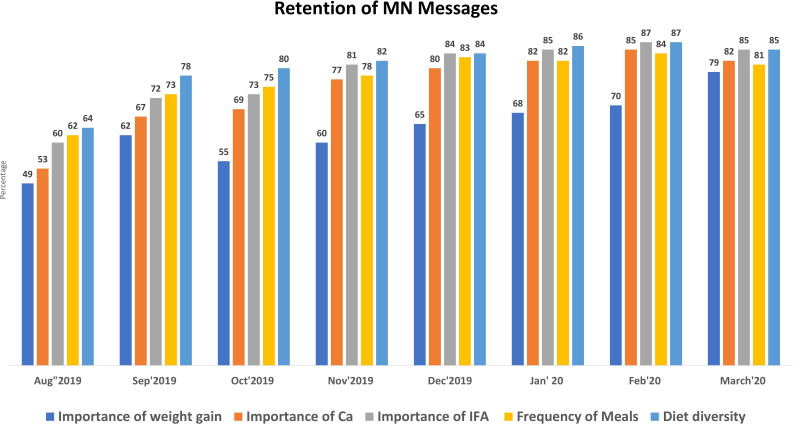
Retention of MN messages. IFA, iron folic acid; MN, maternal nutrition.

Exit interview data showed improvements in the recall of MN messages among pregnant women attending ANC between August and September 2019 (figure 4). Improvements in their recall of the importance of weight gain, calcium tablets, IFA tablets, frequency of meals and diet diversity were observed.

## Lessons and limitations

Multiple changes were tested and applied to improve care in the complex system; QI team learnt to simplify the process by introducing small and doable changes. The improved practices are being institutionalised and sustained through active engagement of the staff and supportive leadership of the department of OBGY. An interdepartmental coordination committee with representation of the departments of OBGY, paediatrics, and PSM under the leadership of the principal to regularly monitor and review progress and provide support as required is critical to sustain the changes.

The hospital continues to face the challenge of ensuring same-day availability of Hb test report to facilitate comprehensive review of nutritional status of antenatal women at OPD by faculty doctor due to limited staff in testing lab. Also, capturing data in MCP card remained a challenge as JRs were involved in other services, and all patients did not reach the ANM who could record the data. Since the hospital is a high-case loadtertiary facility, tracing the PWs in terms of place of delivery was a challenge, which impacted measurement of outcomes for both mothers and newborns.

## Conclusion

**Figure 5:**
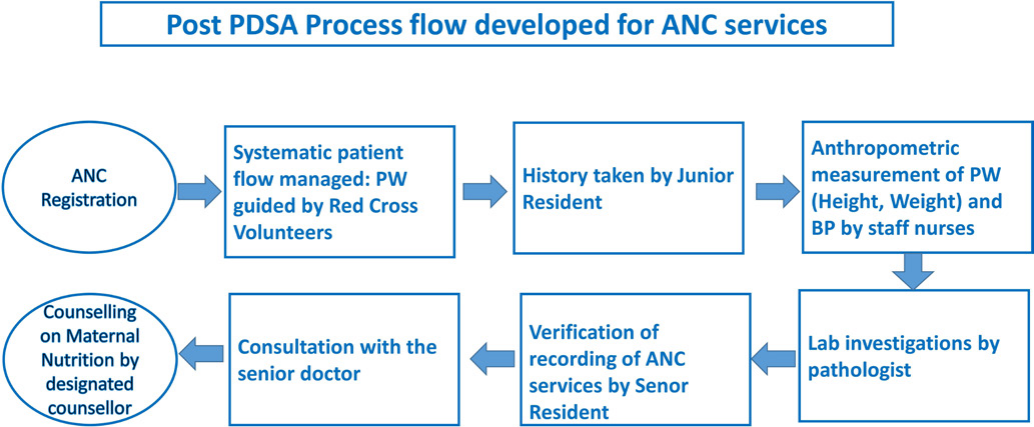
Post PDSA final process flow. ANC, antenatal care; BP, blood pressure; PDSA, Plan–Do–Study–Act; PW, pregnant women.

Successful application of the POCQI approach built confidence and enhanced the motivation of the doctors and nursing staff and provided understanding that service delivery improvements are possible with minimal additional resource investments. It also demonstrated how MN services, including counselling, could be prioritised in ANC and delivered effectively. Moving forward, an attempt to measure and address relevant outcomes of the PWs will provide more authenticity and value for replication. Proactive leadership from the head of the OBGY department, active involvement of the team of doctors and nurses and interdepartmental coordination acted as key drivers of sustainable change. This project could help to improve ANC services in high case load facilities and did not require additional resources. The changes made are sustainable because the processes got institutionalised (figure 5) in the day-to-day delivery system.

## Data Availability

All data relevent to the study are included in the article or uploaded as supplementary information.
